# The dual diversity crisis in EEG biomarker research for cognitive fatigue

**DOI:** 10.1017/S0033291726103808

**Published:** 2026-03-27

**Authors:** Thorsten Rudroff

**Affiliations:** PET Centre, Turku University Hospital, Turku, Finland

Linnhoff et al. present compelling evidence for the frontal aperiodic exponent as a cognitive fatigue biomarker (71% sensitivity, 74% specificity, area under the curve [AUC] = 0.78) (Linnhoff, Cohen Kadosh, & Zaehle, 2026). However, the study exemplifies what I term the “dual diversity crisis”: simultaneous neglect of population diversity (who was studied) and brain diversity (neurobiological heterogeneity within groups). These constitute validity threats that may fundamentally constrain clinical translation.

Beyond gender and age, demographic characterization remains limited – no information on race, ethnicity, socioeconomic status, or education. Recruitment from a single site in Magdeburg, Germany, likely produced a demographically homogeneous sample. If proposed cutoff values derive from such a sample, we cannot assume they generalize to diverse populations. The authors claim “transdiagnostic” value across Multiple Sclerosis (MS) and Long coronavirus disease (COVID), but transdiagnostic ≠ demographically universal. A biomarker performing consistently across two conditions within one population provides no evidence that it will perform equivalently across racial, ethnic, or socioeconomic groups. Previous research demonstrates systematic differences in brain structure and function across demographic groups (Falk et al., 2013; Chiao & Cheon, 2010).

If deployed clinically without proper validation, this biomarker risks systematic misclassification, potentially disadvantaging underserved groups. Moreover, with 119 participants distributed across four groups, statistical power to detect biomarker performance differences across demographic or neurobiological subgroups would be severely limited.

More fundamentally, the study overlooks brain diversity: substantial neurobiological heterogeneity even among individuals with identical symptom profiles. Recent Positron Emission Tomography (PET) neuroimaging provides direct empirical evidence. In a study examining frontal-striatal glucose metabolism in 27 participants (9 MS, 9 Long COVID, and 9 COVID-19 recovered controls) matched for identical fatigue severity scores, identical symptom presentations reflected completely different brain metabolic patterns (Rudroff, 2024): MS patients showed significantly lower frontal glucose metabolism compared to both Long COVID patients and recovered controls; Long COVID patients exhibited significantly higher striatal metabolism than MS patients. These differences persisted despite matching for symptom severity, depression, age, and body mass index, with substantial effect sizes (Cohen’s *d* = 0.89–1.42 for frontal differences, 0.84–1.19 for striatal differences).

These divergent metabolic patterns imply that the Electroencephalogram (EEG) biomarker may be detecting different pathophysiological processes in different individuals – mechanistic pluralism masked by phenomenological similarity. This directly challenges the assumption underlying Linnhoff et al.’s approach that a single biomarker can reliably map onto fatigue regardless of underlying neurobiological heterogeneity. PET neuroimaging demonstrates that individuals experiencing identical cognitive fatigue arrive at that phenomenological endpoint via divergent neural mechanisms – hypometabolism in MS, potentially hypermetabolism in Long COVID.

The convergence of population and brain diversity concerns creates several validity threats. First, mechanistic pluralism: The authors interpret flatter aperiodic slopes as reflecting glutamatergic overload and disrupted E/I balance. However, if identical fatigue symptoms arise from distinct metabolic patterns (as PET demonstrates), the E/I balance captured by EEG may reflect different pathophysiological processes across individuals. A “flatter slope” in one person may represent compensatory hyperexcitation; in another, failed inhibitory control – phenomenologically similar, mechanistically distinct, potentially requiring different therapeutic approaches.

Second, classification performance variability: The reported AUC of 0.78 represents average discriminative ability. Brain diversity predicts that this metric will vary substantially across neurobiological subtypes. Individuals whose fatigue stems from frontal hypometabolism may show excellent biomarker sensitivity; those with preserved frontal function but striatal dysregulation may show poor sensitivity. Without examining performance across neurobiologically defined subgroups, we cannot know if the biomarker works well for everyone or exceptionally well for some and poorly for others.

Third, the symptom score equivalence fallacy: Standardized fatigue scores reflect behavioral outputs, not neural mechanisms. PET data demonstrate that identical scores can reflect opposite metabolic patterns (hypo- vs. hypermetabolism). Using symptom scores as ground truth for biomarker validation assumes neurobiological homogeneity that empirically does not exist.

The authors emphasize transdiagnostic convergence – reduced frontal aperiodic exponents in both MS and Long COVID – as evidence for “overlapping cortical mechanisms” and a “final common pathway.” However, transdiagnostic electrophysiological similarity does not establish mechanistic equivalence. If these conditions reach the same phenomenological endpoint through divergent metabolic pathways, the shared EEG signature may reflect downstream consequences rather than primary pathophysiology. This distinction matters clinically: Interventions targeting glutamate metabolism might benefit individuals with metabolic overactivity but prove ineffective or harmful for those with hypometabolism.

To address the dual diversity crisis in biomarker research, studies should: report comprehensive demographics including race, ethnicity, education, and socioeconomic indicators; conduct stratified analyses examining biomarker performance across demographic and neurobiologically defined subgroups; integrate multimodal neuroimaging (PET, Magnetic Resonance Spectroscopy (MRS), and functional Magnetic Resonance Imaging (fMRI)) to characterize heterogeneity; correlate EEG-derived E/I estimates with direct neurochemical measures; and determine if biomarker-guided treatment selection improves outcomes relative to symptom-based approaches.

The frontal aperiodic exponent represents valuable progress toward objective fatigue assessment. However, simultaneous neglect of population diversity (who was studied) and brain diversity (neurobiological heterogeneity within diagnostic groups) constitutes a dual validity threat that may fundamentally limit clinical translation. PET neuroimaging provides direct evidence that identical symptom presentations arise from divergent brain metabolic patterns, challenging the assumption that any single biomarker can reliably classify fatigue across neurobiologically diverse individuals.

Until we validate proposed biomarkers across both population and brain diversity, we risk systematically excluding patients who might benefit most from objective assessment tools. The goal should not be abandoning biomarker research but pursuing it with methodological rigor matching clinical ambitions.
